# PB.49: Preoperative axillary staging in breast cancer patients: repeat sampling in cases of suspicious ultrasound or inconclusive cytology

**DOI:** 10.1186/bcr3549

**Published:** 2013-11-08

**Authors:** L Clarke, P Newton, A Leaver

**Affiliations:** 1 Queen Elizabeth Hospital, Gateshead, UK

## Introduction

In our Trust preoperative axillary staging comprises ultrasound with appearances scored LN1 to LN5, and fine needle aspiration (FNA) of LN3 to LN5 nodes. Repeat sampling with FNA or core biopsy (CB) is performed on inconclusive FNA (C1, C3, C4) at the discretion of the radiologist. Repeat sampling is also performed in LN4/5 C2 patients. There has been a trend toward the more invasive and expensive CB for repeat axillary sampling in our unit. Here we assess our practice and the diagnostic yield from repeat sampling, to review current departmental guidelines.

## Method

All invasive breast cancer patients who underwent surgery in 2012 were identified from multidisciplinary meeting records. Cytology and histology reports from repeat preoperative sampling and surgery were reviewed.

## Results

In 2012, 169 female breast cancer patients underwent axillary FNA and axillary surgery. FNA was followed by CB in 26% (44/169) and no FNAs. Of C1/C3/C4 FNAs, 34/39 had CB. Of these, 10/34 were malignant. CB was negative in 23, with 20/23 negative nodes and 3/23 positive nodes at surgery. There were 79 C2 FNAs with 10/79 undergoing CB. Nine of 10 cores were negative, of which one was node-positive at surgery. The 1/10 positive core represented micrometastasis on clearance. No complications resulted from axillary FNA or CB.

## Conclusion

Repeat biopsy including CB is useful in C1/C3/C4 cytology, improving triage to the correct axillary surgical procedure. Repeat biopsy of C2 nodes with LN4/5 ultrasound has not been validated here: ongoing audit and a higher threshold for repeat biopsy in these patients should be considered.

